# Striatum Shape Hypertrophy in Early Stage Parkinson’s Disease With Excessive Daytime Sleepiness

**DOI:** 10.3389/fnins.2019.01353

**Published:** 2020-01-09

**Authors:** Liang Gong, Huaisu Li, Dan Yang, Yinwei Peng, Duan Liu, Ming Zhong, Bei Zhang, Ronghua Xu, Jian Kang

**Affiliations:** ^1^Department of Neurology, Chengdu Second People’s Hospital, Chengdu, China; ^2^Hospital of Chengdu University of Traditional Chinese Medicine, Chengdu, China

**Keywords:** Parkinson’s disease, excessive daytime sleepiness, striatum, shape analysis, structure

## Abstract

**Introduction:**

Excessive daytime sleepiness (EDS) is one of the common and burdensome non-motor symptoms of Parkinson’s disease (PD). However, the underlying neuropathology mechanism in PD patients with EDS (PD-EDS) remains unclear. The present study aims to delineate potential locations of structural alteration of subcortical regions in early stage and drug-naïve PD-EDS.

**Methods:**

The study had 252 patients with PD and 92 matched healthy controls (HC). EDS was estimated with the Epworth Sleepiness Scale, with a cutoff of 10. Ultimately, 59 patients were considered as PD-EDS. The remaining 193 were PD patients without EDS (PD-nEDS). FMRIB’s Integrated Registration and Segmentation Tool (FIRST) was employed to assess the volumetric and surface alterations of subcortical nuclei in PD and PD-EDS.

**Results:**

Volumetric analyses found no difference in the subcortical nucleus volume between PD and HC, or PD-EDS and PD-nEDS groups. The shape analyses revealed the local atrophic changes in bilateral caudate and right putamen in patients with PD. In addition, the hypertrophic changes were located in the right putamen and left pallidum in PD-EDS than in PD-nEDS.

**Conclusion:**

Our findings revealed the regional hypertrophy of the striatum in PD-EDS. Our results indicate that local hypertrophic striatum would be a valuable early biomarker for detecting the alteration in PD-EDS. The shape analysis contributes valuable information when investigating PD-EDS.

## Introduction

Parkinson’s disease (PD) is the second most common neurodegenerative disorder associated with both motor and numerous non-motor symptoms (De Lau and Breteler, 2006). Excessive daytime sleepiness (EDS), a non-motor feature, is described as inappropriate and undesirable sleepiness during waking hours, affecting 16–50% of patients with PD (Knie et al., 2011). As EDS has a significant negative impact on the quality of life and driving safety (Meindorfner et al., 2005; Chahine et al., 2017), it is quite essential to fully understand the neurobiological mechanism underlying this symptom.

Although studies have shown associations with EDS symptoms in PD including non-tremor dominant phenotype, autonomic dysfunction, depression, anxiety, and disorders of rapid eye movement sleep behavior (Simuni et al., 2015; Amara et al., 2017; Wen et al., 2017), the neural mechanism of EDS in PD remains poorly understood. The lack of correlation between EDS and disease severity of PD [Hoehn & Yahr stage (H&Y)] has led to the notion that the EDS might be associated with PD-specific pathology (Yousaf et al., 2018b). However, *in vivo* neuroimaging quantification has been used to detect early pathophysiological changes in PD with EDS (PD-EDS), potentially serving as a biomarker for disease progression and treatment monitoring (Chondrogiorgi et al., 2016; Wen et al., 2017; Ashraf-Ganjouei et al., 2019). Molecular imaging studies using positron emission tomography (PET) and single-photon emission computed tomography (SPECT) implicate EDS with dopaminergic dysfunction in subcortical regions (Happe et al., 2007; Pagano et al., 2016). However, molecular imaging is expensive and radioactive, so it would not be a conventional and regular scan. T1-weighted magnetic resonance imaging (MRI) is one of the most widely used sequences in neuroimaging studies; it can be acquired in all scanners and is commonly used in conventional clinical MRI protocols. Presently, three studies have used the whole-brain gray matter analysis and revealed widespread volume reductions in the frontal, occipital, temporal, and limbic lobes in PD patients with EDS (PD-EDS) (Gama et al., 2010; Kato et al., 2012; Chondrogiorgi et al., 2016). However, for the subcortical nucleus, these studies have contradictory findings, reporting both increased and decreased gray matter volumes (GMs) in the hippocampus and parahippocampus in patients with PD-EDS. One reason for inconsistent findings might be the small sample size and antiparkinsonian medication in previous studies. Another important factor might be attributed to the limitation of the voxel-based morphometry (VBM) analysis, which is based on a standard template. A previous volumetric analysis of PD pathology also showed conflicting results on subcortical structures (Uitti et al., 2005; Gama et al., 2010; Péran et al., 2010).

Several surface-based subcortical region shape analyses of PD patients have revealed local atrophy in the subcortical nucleus, including the caudate nucleus and the putamen, and a correlation between cognitive function and atrophy of the caudate nucleus and the putamen (Apostolova et al., 2010; Sterling et al., 2013; Menke et al., 2014; Nemmi et al., 2015). Recently, Nemmi et al. (2015) showed that the shape analysis was the most sensitive method for observing atrophy-related differences between PD patients and control subjects. They also found that the information from the shape analysis was able to discriminate PD patients from healthy control subject best when compared with the standard volumetric and gray matter density analysis (Nemmi et al., 2015). Thus, we hypothesize that the shape analysis could be a useful tool to detect localized subcortical nuclei alterations in PD-EDS.

To test our hypothesis, we used a fully automated segmentation method (FIRST, Oxford Centre, FMRIB) and the replicable vertex-based shape analysis in our study. We compared the subcortical nuclei shape difference between early stage and drug-naïve PD patients and matched healthy controls (HC). The PD patients group was further divided into PD-EDS and PD without EDS subgroups (PD-nEDS) according to the Epworth Sleepiness Scale (ESS; with a cutoff of 10), and the shape difference between these two PD groups was conducted in each subcortical nucleus, separately. We also performed a traditional volumetric-based analysis between groups.

## Materials and Methods

### Participants

All participants were enrolled in PPMI (Parkinson’s Progression Markers Initiative), an observational, international, multicenter investigation of clinical, biological, and neuroimaging markers of PD progression, where all patients with PD were newly diagnosed and untreated at baseline (Marek et al., 2011). Study aims, methodology, and details of study assessments are available on the PPMI website^1^. The inclusion and exclusion criteria were described previously in detail (Wen et al., 2016; Chahine et al., 2019). Institutional review boards approved the study at PPMI sites, and written informed consent was obtained.

As of August 4, 2019, the participants in the PPMI database include 454 patients and 215 matched HC. Only participants with T1 structural MRI data and an ESS assessment were included in the present study; 350 participants were selected for MRI processing, and six were excluded based on image-processing quality control (poor segmentation). The final sample included 252 patients with PD and 92 HC subjects.

### Assessments and Subgroup of Parkinson’s Disease

The ESS was used for EDS evaluation; this scale has high test–retest correlation and high internal consistency (Johns, 1991). ESS is recommended for assessing and measuring the severity of EDS in PD by the Movement Disorders Society Sleep Scale Task Force (Högl et al., 2010). According to the ESS cutoff score recommend, patients with PD were categorized as having EDS (PD-EDS group) if ESS was equal or above 10 and not having EDS (PD-nEDS group) if ESS score was lower than 10 (Arnulf et al., 2002; Matsui et al., 2006; Högl et al., 2010; Amara et al., 2017). As indicated by the ESS cutoff score, 192 PD patients were subdivided to PD-EDS group, and 53 PD patients were subdivided to PD-nEDS group. The Movement Disorders Society Unified Parkinson’s Disease Rating Scale score (UPDRS) part III was used to measure motor function (Goetz et al., 2008), and the Montreal Cognitive Assessment (MoCA) was used to test global cognitive function (Nasreddine et al., 2005). The 15-item Geriatric Depression Scale (GDS) to test mood symptoms (Weintraub et al., 2006) and the Rapid Eye Movement Sleep Behavior Disorder Screening Questionnaire (RBDSQ) was selected as a measure of rapid eye movement sleep behavior disorder (RBD) (Stiasny–Kolster et al., 2007).

### MRI Acquisition

The MRI acquisition procedures were displayed in detail at http://www.ppmi-info.org/wp-content/uploads/2017/06/PPMI-MRI-Operations-Manual-V7.pdf. In brief, a three-dimensional (3D), T1-weighted sequence (e.g., MPRAGE or SPGR) is required. The field of view (FOV) must include the vertex, cerebellum, and pons. The T1-weighted image must be acquired as a 3D sequence and have a slice thickness of 1.5 mm or less with no interslice gap. The PPMI core optimized the acquisition sequence across sites to minimize bias in data between sites and maximize comparability of data in the study. Typical MRI parameters were as follows: repetition time 5–11 ms; echo time 2–6 ms; thickness 1.2 mm; gap 0 mm; voxel size 1 × 1 × 1.2 mm; matrix 256 × 256 × 170–200.

### Image Preprocessing

MRI data analyses were performed using the tools from FSL (version 5.0.9^2^; FMRIB Software Library, Oxford University, Oxford, United Kingdom) (Jenkinson et al., 2012).

First, the SIENAX^3^ was used to estimate the total intracranial volume (eTIV), white matter volume (WM), and GM for all the subjects. All reported brain volumes were normalized to a “normalized” skull size (Smith et al., 2002).

Second, the subcortical structures were segmented using the FMRIB’s Integrated Registration and Segmentation Tool (FIRST^4^, part of FSL, version 5.0.9) (Patenaude et al., 2011). FIRST is an automated tool to segment the subcortical nuclei and has been used to study several neuropsychiatric disorders (van den Bogaard et al., 2011; Seifert et al., 2015).

Third, after the automated segmentation (fun_first_all), the quality of segmentation for each subject was checked manually (first_roi_slicesdir). The outcome file of FIRST was then used for the volume and vertex analysis. For the standard volumetric analysis, the raw volume subcortical structure was normalized for the inter-individual variability of brain size (raw volume/eTIV).

### Statistical Analysis

A two-sample *t*-test was conducted to compare various demographic data between the two groups, whereas the chi-squared test was used to compare sex and H&Y stage. An analysis of covariance (ANCOVA) was used to estimate the group differences in the whole brain volume (eTIV) and normalized subcortical structure volume, with age, sex, and eTIV (not in the eTIV comparison) as covariates (SPSS 20, Inc., Chicago, IL, United States). Pearson correlation was employed to examine the relationship between ESS, MoCA, GDS, and EBDSQ scores in the PD group. Statistical significance was set at *p*-values < 0.05, after correction for multiple comparisons using the false discovery rate (FDR).

### Surface-Based Shape Analysis

The new version of vertex-wise analysis was employed to investigate localized shape differences in the subcortical nucleus between HC and PD, as well as the group differences between PD-EDS and PD-nEDS, separately. The shape analyses were all adjusted for age, sex, and eTIV (first_utils and randomize, FSL 5.0.9). This approach calculates the group differences on a per-vertex basis. The threshold-free cluster enhancement (TFCE), a new method for finding significant “clusters” in the statistic image without having to define clusters in a binary way, was used for multiple comparison correction (Smith and Nichols, 2009). As the traditional surface-based vertex analysis comprises the vectors in each significant vertex, we used it to display the direction of group differences.

## Results

### Demographic and Behavioral Features

Detailed subject characteristics and clinical parameters for each group are summarized in Table 1. There are no significant differences in age, sex, education, ESS score, GDS score, or eTIV between PD and HC groups. The EBDSQ score was higher and MoCA score was lower in the PD group than in the HC group. Similarly, except the difference in ESS, there are no intergroup differences in age, sex, education, disease duration, age of onset, H&Y stage, UPDRS-III, MoCA, GDS, EBDSQ score, or eTIV between PD-nEDS and the PD-EDS groups.

**TABLE 1:** Demographic and clinical characteristics of all participants.

**Subject groups**	**HC**	**PD**	**PD-EDS**	**PD-nEDS**	***p***-**value (HC vs. PD)**	***p***-**value (PD-EDS vs. PD-nEDS**
***N***	**92**	**252**	**59**	**193**		
Age (years)	59.81 ± 10.44	61.34 ± 9.51	62.23 ± 8.91	60.54 ± 10.05	0.203	0.361
Gender (female/male)	30/62	96/156	25/34	70/123	0.210^†^	0.445^†^
Education (years)	15.78 ± 2.88	15.31 ± 3.14	15.83 ± 3.10	16.01 ± 3.00	0.216	0.763
Disease duration (months)	–	6.80 ± 7.44	7.19 ± 7.29	6.61 ± 7.44	–	0.682
Onset (years)		59.27 ± 10.07	60.52 ± 8.83	58.26 ± 10.76	–	0.249
H&Y (1/2)		118/134	23/36	95/98	–	0.285^†^
UPDRS-III	0.54 ± 1.22	19.60 ± 9.17	21.34 ± 10.67	18.62 ± 8.40	<0.001	0.129
MoCA	28.30 ± 1.30	27.14 ± 2.42	27.06 ± 2.81	27.48 ± 2.20	<0.001	0.376
ESS	6.71 ± 3.67	6.99 ± 3.57	12.08 ± 2.18	5.44 ± 2.21	0.510	<0.001
GDS	5.25 ± 1.55	5.32 ± 1.43	5.35 ± 2.01	5.35 ± 1.43	0.673	0.993
EBDSQ	3.77 ± 2.28	5.55 ± 2.77	5.67 ± 3.09	5.08 ± 2.86	<0.001	0.178
eTIV	1,525.98 ± 593.29	1,534.63 ± 564.14	1,383.31 ± 497.73	1,518.73 ± 534.80	0.900	0.178

*All values are presented as means and standard deviation (SD). ^†^*p-*value was calculated using chi-squared test. HC, healthy control; PD, Parkinson’s disease; EDS, excessive daytime sleepiness; PD-EDS, PD patients with EDS; PD-nEDS, PD patients without EDS; H&Y, Hoehn–Yahr stage; UPDRS-III, Unified Parkinson’s Disease Rating Scale part III; MoCA, Montreal Cognitive Assessment; ESS, Epworth Sleepiness Scale; GDS, Geriatric Depression Scale; EBDSQ, rapid eye movement episode sleep behavior disorder (RBD) screening questionnaire; eTIV, estimated total intracranial volume.*

The correlation analyses revealed that the EDS scores were significant and positively correlated with EBDSQ scores (*r* = 0.223, *p* = 0.004) and GDS scores (*r* = 0.126, *p* = 0.046) in the PD group. However, the relationship between EDS and GDS was not significant after FDR correction. The EDS scores did not correlate significantly with cognitive function (MoCA) and motor symptom (UPRDS-III) in the PD group. In addition, the EDS score in patients with H&Y stage 2 was significantly higher than that in H&Y stage 1 (7.43 vs. 6.47, *p* = 0.03).

### Subcortical Nuclei Global Normalized Volume Comparison

There was no significant difference in any of the subcortical nucleus volumes between PD and HC and between PD-EDS and PD-nEDS groups in the global normalized volume of each nucleus after FDR correlation (Table 2).

**TABLE 2:** Mean normalized volume of subcortical structure among groups.

**Subcortical region**	**HC (*n* = 92)**	**PD (*n* = 252)**	***t*-value**	***p*-value**	**PD-EDS (*n* = 59)**	**PD-nEDS (*n* = 193)**	***t*-value**	***p***-**value**
Left accumbens	0.38 ± 0.15	0.36 ± 0.17	0.86	0.39	0.35 ± 0.14	0.38 ± 0.14	−1.10	0.27
Left amygdala	1.10 ± 0.39	1.09 ± 0.40	0.11	0.91	1.07 ± 0.33	1.20 ± 0.39	−1.86	0.07
Left caudate	2.48 ± 0.85	2.51 ± 0.88	–0.28	0.78	2.46 ± 0.78	2.68 ± 0.76	−1.46	0.15
Left hippocampus	2.80 ± 1.01	2.83 ± 1.00	–0.30	0.76	2.80 ± 0.86	3.10 ± 0.97	−1.75	0.08
Left pallidum	1.33 ± 0.47	1.38 ± 0.48	–0.79	0.43	1.33 ± 0.42	1.45 ± 0.42	−1.56	0.12
Left putamen	3.54 ± 1.22	3.61 ± 1.27	–0.45	0.65	3.57 ± 1.11	3.78 ± 1.09	−1.03	0.31
Left thalamus	5.96 ± 2.06	6.03 ± 2.04	–0.26	0.79	6.00 ± 1.78	6.41 ± 1.79	−1.18	0.24
Right accumbens	0.30 ± 0.12	0.30 ± 0.14	0.26	0.80	0.30 ± 0.13	0.30 ± 0.11	−0.02	0.99
Right amygdala	1.11 ± 0.41	1.16 ± 0.42	–0.98	0.33	1.13 ± 0.33	1.25 ± 0.36	−1.93	0.06
Right caudate	2.54 ± 0.89	2.60 ± 0.89	–0.52	0.60	2.54 ± 0.76	2.81 ± 0.81	−1.85	0.07
Right hippocampus	2.89 ± 1.04	2.89 ± 1.03	–0.01	0.99	2.87 ± 0.92	3.05 ± 0.87	−1.01	0.32
Right pallidum	1.36 ± 0.48	1.39 ± 0.48	–0.43	0.67	1.37 ± 0.43	1.45 ± 0.41	−1.08	0.28
Right putamen	3.59 ± 1.25	3.66 ± 1.29	–0.41	0.68	3.61 ± 1.11	3.85 ± 1.09	−1.12	0.27
Right thalamus	5.83 ± 1.99	5.92 ± 2.00	–0.34	0.73	5.93 ± 1.77	6.25 ± 1.74	−0.93	0.35

*The values represent the mean and standard deviation of the ratio between structures’ volume and eTIV. eTIV, estimated total intracranial volume.*

### Shape Comparisons of Parkinson’s Disease and Control Subcortical Structures

**FIGURE 1:**
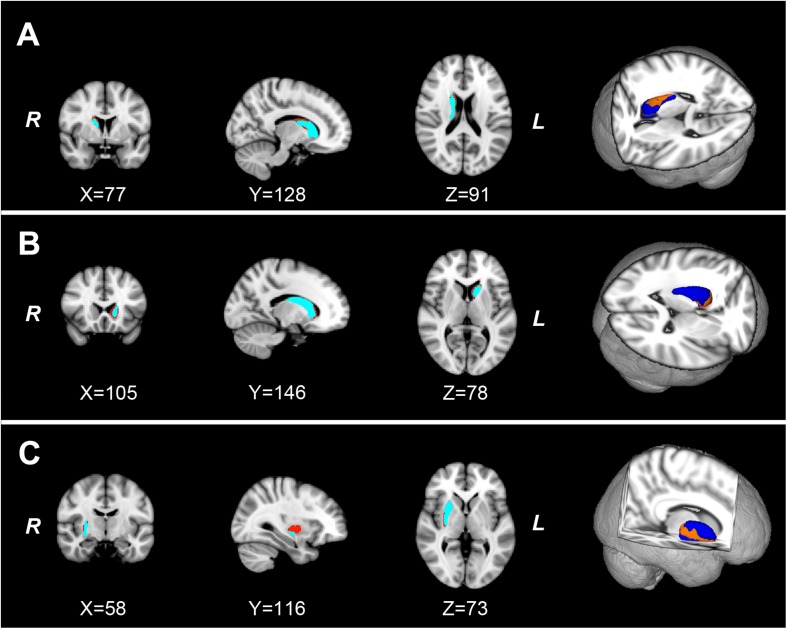
The localized shape differences between HC and PD groups using vertex-wise surface analyses of each subcortical nucleus. The regions in orange indicate the different regions of the characteristic subcortical nuclei between PD and HC groups. **(A)** Group differences of the right caudate are located in the body and tail subdivisions. **(B)** Group differences of the left caudate are located in the head subdivision. **(C)** The group differences of the right putamen are located in the ventrolateral subdivision of putamen. HC, healthy control; PD, Parkinson’s disease.

**FIGURE 2:**
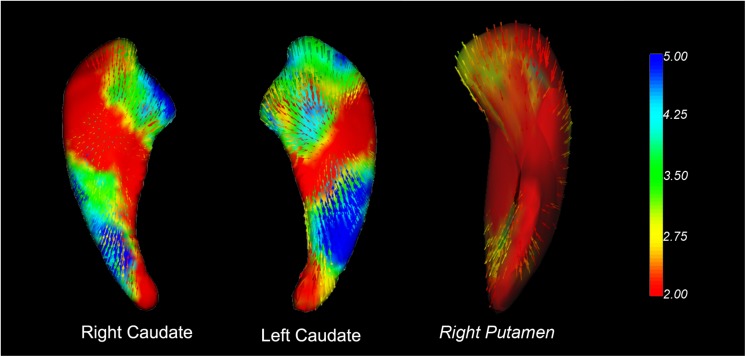
Vector graphs of the bilateral caudate and right putamen according to the traditional surface-based vertex analysis displayed by 3D mesh. The color bar indicates the statistical values; an increase from red to blue indicates a lower to higher statistical significance. The small arrows shown on the surface indicate the direction of change. The inward arrows indicate the direction of difference, suggesting that these subcortical nuclei are smaller/thinner here than in the healthy control groups.

As shown in Figure 1, the new vertex analysis revealed that the body and right tail caudate, the left head caudate, and the right ventrolateral putamen showed significant group differences in the PD group than in the HC group (TFCE corrected). The traditional surface-based vertex analysis showed an inward displacement in these significantly different regions of the bilateral caudate and right putamen (Figure 2), whereas the findings of shape analysis indicated a localized caudate and putamen volume atrophy in the PD group than in the HC group. No significant areas of hypertrophy were observed. No significant group differences were found in the shape analysis of the other subcortical nuclei.

### Shape Comparisons of Parkinson’s Disease With Excessive Daytime Sleepiness and Parkinson’s Disease Without Excessive Daytime Sleepiness Subcortical Structures

**FIGURE 3:**
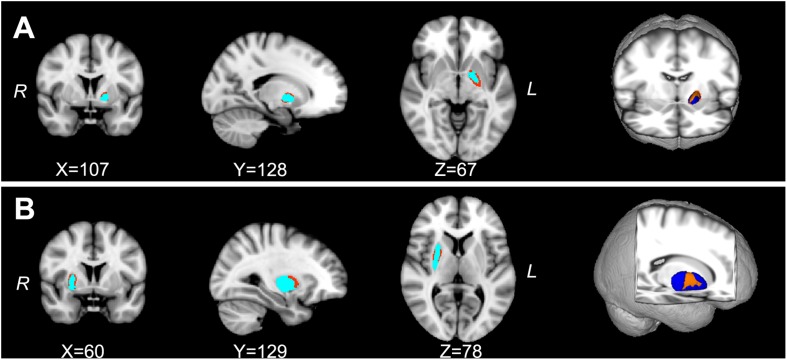
The localized shape differences between PD-EDS and PD-nEDS subgroups using vertex-wise surface analyses of each subcortical region. The regions in orange indicate the different regions of the special subcortical nuclei between PD-EDS and PD-nEDS groups. **(A)** The group differences of the left pallidum are located in the left dorsolateral subdivision of pallidum; **(B)** the group differences of the right putamen are located in the middle subdivision of putamen. PD-EDS, Parkinson’s disease with excessive daytime sleepless; PD-nEDS, Parkinson’s disease without excessive daytime sleepless.

**FIGURE 4:**
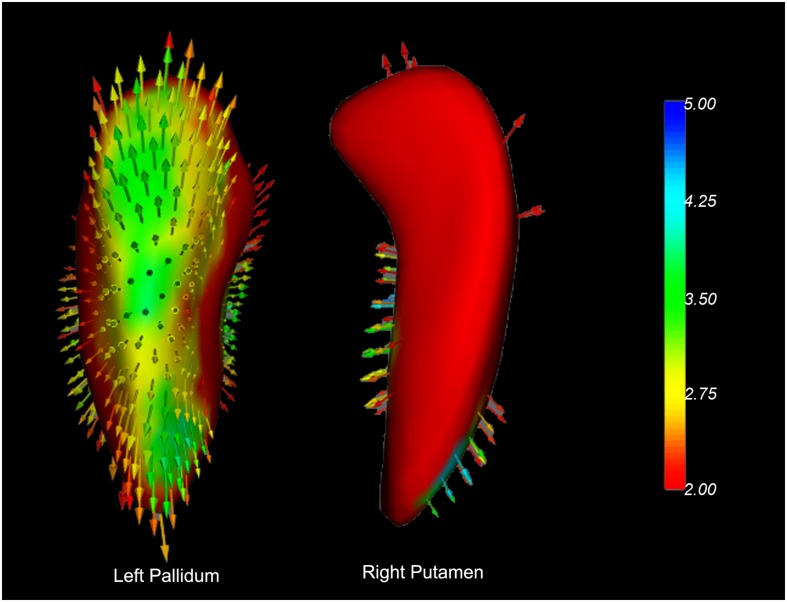
Vector graphs of the bilateral caudate and right putamen according to the traditional surface-based vertex analysis displayed by 3D mesh. The color bar indicates the statistical values; an increase from red to blue indicates a lower to higher statistical significance. The small arrows shown on the surface indicate the direction of change. The inward arrows indicate the direction of difference is such that these subcortical nuclei are smaller/thinner here than in the healthy control groups.

The shape analyses also revealed significant group differences in the left dorsolateral pallidum and the right dorsal putamen between the PD-EDS and PD-nEDS groups (Figure 3). The traditional surface-based vertex analysis showed an outward displacement in these significantly different regions of pallidum and putamen (Figure 4); thus, the shape analysis results indicate a localized pallidum and putamen volume hypertrophy in PD-EDS than in PD patients without EDS (PD-nEDS). No significant group differences were found in the shape analysis of the other subcortical nuclei.

## Discussion

The current study employed surface-based shape analysis to investigate the spatial distribution change of subcortical nuclei in drug-naïve early stage patients with PD and PD-EDS. This study has two main findings: First, we verified that the atrophy of striatum volume is not global but regional in the patients with PD. Specifically, the regional atrophy in PD was located in the right tail caudate nuclei, left head caudate nuclei, and right ventrolateral putamen. Second, the PD-EDS showed regional hypertrophic volume alteration in the striatum when compared with PD-nEDS. The hypertrophied striatum was located in the left dorsolateral pallidum and right dorsal putamen. To our knowledge, this is the first study demonstrating PD-EDS-related shape differences in the striatum. Our findings indicate that the region-specific striatum shape alteration would be the early biomarker in PD-EDS.

The results of the present study are consistent with previous reports of striatal shape atrophy in patients with PD and indicate that striatal shape alteration between PD and control subjects are most robust in the caudate nuclei and putamen (Apostolova et al., 2010; Sterling et al., 2013; Nemmi et al., 2015). Previous studies used manual, semi-automated, and machine learning approaches for subcortical structure region segmentation and shape analysis (Apostolova et al., 2010; Pitcher et al., 2012; Sterling et al., 2013). Apostolova et al. (2010) reported that PD with dementia showed atrophy in the left medial and lateral and right medial of the caudate. Sterling et al. (2013) found that the most significant atrophic putamen in PD was localized in the caudal and ventrolateral areas, and the most atrophic caudate was located at the rostral caudate head. They also reported the association between cognition performance and the altered region of putamen (Sterling et al., 2013). The local putamen atrophy would attribute to the reduced dopaminergic activity and striatal dopamine depletion spine loss in the putamen in early disease stages of PD (Geng et al., 2006; Burguière et al., 2013; Sterling et al., 2013). In addition, the atrophied caudate nuclei were located in the right tail and left head region. The results indicate a hemispheric difference in PD patients, supporting the notion of an endogenous, inter-hemispheric dopamine imbalance in the mesostriatal dopaminergic system (Molochnikov and Cohen, 2014).

The existing neuroimaging studies on the EDS symptom in PD patients have found EDS-related alteration at brain structural, functional, and metabolic levels (for review, see Yousaf et al., 2018b). PD-EDS showed dopamine transporter (DAT) uptake reduction in the caudate, which correlated with clinical EDS symptom (Yousaf et al., 2018a). Happe et al. (2007) reported that the DAT binding in the striatum and putamen inversely correlates with EDS score in early PD. To our knowledge, no neuroimaging study focused on the structural shape alteration of the striatum in PD-EDS. In our study, the hypertrophic alteration in the left dorsolateral pallidum and right dorsal putamen was found in PD-EDS than in PD without EDS. The results are consistent with previous structural studies on sleep disorders. Hypertrophic cortical and subcortical alterations have been reported in other sleep disorders, such as obstructive sleep apnea and primary insomnia (Rosenzweig et al., 2013; Baril et al., 2017; Yu et al., 2018). The hypertrophic structural change of the striatum in the sleep disorders implied the intricate endogenous repair systems in the brain, and the preconditioning and enhanced neurogenesis mechanism might be included (Lledo et al., 2006; Dirnagl et al., 2009). EDS also impacts the striatum in the early stage of PD. Our study supports the notion that EDS might be a preclinical marker of PD (Arnulf et al., 2002), and the EDS symptom in the drug-naïve and early stage PD might be attributed to the compensatory mechanism of the striatum. Further studies with longitudinal designs are warranted to clarify whether the compensation will reverse to maladaptation during the disease progression.

It is interesting to note that the normalized global volume of the subcortical nuclei did not show a significant difference for any of the structures between the PD and HC, PD-EDS, and PD-nEDS groups. These findings are consistent with previous observations on patients with PD where volume did not show any difference between the PD and HC groups, but the shape analysis was able to detect the significant difference (McKeown et al., 2008; Apostolova et al., 2010; Nemmi et al., 2015; Tanner et al., 2017). Several studies have found decreased subcortical nuclei volumes in the putamen, thalamus, and hippocampus in the PD group than in the HC group; the PD patients recruited in these studies were in the late stage of the of disease, were at mild stage, have dementia, and undergoing dopaminergic treatment (Halliday, 2009; Pitcher et al., 2012; Nemmi et al., 2015; Tanner et al., 2017). These findings all suggested that the surface-based shape analysis would be more sensitive to detect the early change of subcortical structures in patients with PD and in PD-EDS.

There are several limitations to our study. First, there are no objective measures of EDS in the present study, and the subjective assessment may result in underestimation of this symptom (Kaynak et al., 2005). Second, structural association of the striatum with EDS in PD patients could not be explained as a causal relationship, and further longitudinal studies would be necessary to confirm the hypertrophic shape change as dynamic components of the progression in PD-EDS. Third, the PD patients were all in the early stage, and the severity of EDS in our group is moderate (below 16). We proposed that the hypertrophic alteration in striatum might be a compensatory mechanism in the mild severity of EDS in PD patients. Further studies should enroll severe EDS patients to verify our speculation. Lastly, all patients in our study were drug naïve; however, another cause of EDS is drug therapy, including dopamine agonists and levodopa (Knie et al., 2011). How the striatum structural alteration in PD with the EDS occurred after treatment with dopaminergic agents should be investigated in future studies.

## Conclusion

In summary, the present study verified the localized atrophic striatum in patients with PD. In addition, we found the regional putamen and pallidum hypertrophy in PD-EDS. Our results indicate that compensatory mechanisms might be involved in the early stage of PD-EDS, and the shape alteration of stratum would be a useful biomarker for early detection in the PD-EDS.

## Data Availability Statement

Publicly available datasets were analyzed in this study. This data can be found here: www.ppmi-info.org/data.

## Author Contributions

LG and JK designed the study. LG, HL, DY, and JK analyzed and drafted the manuscript. YP, MZ, DL, BZ, and RX made substantial contribution to the data interpretation, critically revised, and drafted the manuscript. All the authors read and approved the final version of the manuscript.

## Conflict of Interest

The authors declare that the research was conducted in the absence of any commercial or financial relationships that could be construed as a potential conflict of interest.

## Footnotes

1 http://www.ppmi-info.org/study-design

2 https://fsl.fmrib.ox.ac.uk/fsl

3 https://fsl.fmrib.ox.ac.uk/fsl/fslwiki/SIENA

4 https://fsl.fmrib.ox.ac.uk/fsl/fslwiki/FIRST
